# A Case of Painless Labor

**Published:** 1875-05

**Authors:** Swan M. Burnett

**Affiliations:** Knoxville, Tenn.


### A CASE OF PAINLESS LABOR.

BY SWAN M. BURNETT, M. D., KNOXVILLE, TENN.

Dr. J. W. Thornburgh, of New Market, in this State, has communicated to me, a case of painless labor, which I think of sufficient interest to present to the readers of the Record.

Mrs. Fennell, of Granger county, sent for him one evening at about 5 o'clock, to attend her in confinement. After his arrival, he warmed himself at the fire and naturally expected to hear some manifestations of labor, but hearing none after the lapse of some time, he went to the bed, and asked her how she was getting on. She replied quite well, she supposed, though she was suffering no pain. He then asked her how she knew she was in labor? She answered that the water had broken, and that she had always regarded as a sign that labor had begun. The Doctor then made an examination *per vaginam* and found the mouth of the womb considerably dilated. He questioned her again as to pain, and she was quite offended that he should doubt her statement as to perfect freedom from pain. He watched her closely, and made frequent examinations, but was never able to observe the least evidence of pain, or any intermittent contraction of the uterus. From hour to hour he noticed a steady advance in the head, but no alternate advance and retrogression such as we usually observe in parturition.

At about ten o'clock the child was born whilst she was sitting on another woman's lap quietly smoking a pipe. There were no after-pains, and she made a good recovery.

Cases of very rapid labor, with trifling pain are common, but I know of no case on record of a painless labor of five hours duration, and what is of peculiar interest, the labor seemed to be effected entirely by a tonic contraction of the uterus. She was the mother at that time of three children, in giving birth to which she had suffered the usual pains of parturition. Dr. Thornburgh subsequently attended her in a normal labor of about the length of this one, and she suffered as much as women ordinarily do. She has also since given birth to a child in the same painless manner as in this reported instance, and was attended by a midwife still living in Granger county.

It is an important question in the physiology of parturition, why n these two instances was labor perfectly painless, while in the others the usual amount of pain was present?—and what is the connection between the painless labor and the unintermittent contraction of the uterus?

---

SIXTEEN cases of trichina, from eating diseased pork, are reported at Kankakee, Ill. Two of the victims have died, and several others are dangerously ill.—*Med. and Surg. Reporter.*

